# Should intraoperative nerve monitoring be used routinely in primary thyroid surgeries?

**DOI:** 10.12669/pjms.36.2.1054

**Published:** 2020

**Authors:** Murat Akici, Murat Cilekar, Sezgin Yilmaz, Yuksel Arikan

**Affiliations:** 1 Dr. Murat Akici, Department of General Surgery, Afyonkarahisar Health Sciences University, Afyon, Turkey; 2 Dr. Murat Cilekar, Department of General Surgery, Afyonkarahisar Health Sciences University, Afyon, Turkey; 3 Dr. Sezgin Yilmaz, Department of General Surgery, Afyonkarahisar Health Sciences University, Afyon, Turkey; 4 Dr. Yuksel Arikan, Department of General Surgery, Afyonkarahisar Health Sciences University, Afyon, Turkey

**Keywords:** Intraoperative, Nerve monitoring, Thyroid, Nerve paralysis, Goitre

## Abstract

**Objective:**

This study presents the effects of intraoperative nerve monitoring on RLN injuries in patients who underwent primary surgery for benign thyroid pathology.

**Methods:**

We retrospectively evaluated the data of 273 patients who had primary thyroidectomy due to benign thyroid pathology between January 2012 and July 2017. The patients were classified into two groups. Group-1 consists of patients whose nerves were monitored. We separated the patients whose nerves were not monitored into Group-2.

**Results:**

There were 140 and 133 patients in Groups 1 and 2, respectively. Regarding the age, gender and surgical indication between the groups, statistically significant difference was not found (P > 0.05). In Group-1, transient paralysis developed in four patients (2.9%). The permanent paralysis developed in one patient (0.7%). In Group-2, transient paralysis developed in nine patients (6.8%). The permanent paralysis developed in four patients (3%). When the groups were evaluated, there was statistically significant difference in terms of transient and permanent paralysis (P=0.01, P =0.001, respectively).

**Conclusions:**

In view of the negative effects of RLN injury on the patient, we think that intraoperative nerve monitoring should be used routinely in benign thyroid surgeries.

## INTRODUCTION

Thyroidectomy is one of the most frequently performed operations in iodine-deficient regions. Recurrent laryngeal nerve (RLN) injury and hypoparathyroidism are the prevalent complications during thyroidectomy.^1^ Risk of transient vocal cord paralysis (VCP) (2%-5.1%) is higher than RLN injury risk (0.6%-1%) during a thyroid surgery.^2^ RLN paralysis risk varies between 0.5% and 20% depending on the disease type (benign or malign), surgery type (primary or secondary), resection width, and surgical technique.^3^-^5^ Existence of anatomic variation, large gland, and inexperienced surgeon are the additional risk factors of RLN paralysis.^3^ Dissection of RLN and its visibility along the whole trace are the gold standard method to prevent RLN injuries during a thyroid surgery.^6^

Viewing the nerve during the surgery protects its structural integrity. However, the anatomical integrity does not provide information about the nerve’s functional continuity and postoperative function of the vocal cord. Main reason of this in a thyroidectomy operation is not cutting but stretching the nerve.^3^,^7^-^9^ Intermittent intraoperative nerve monitoring (I-IONM) is the standard method to evaluate the nerve function by stimulating RLN and N.Vagus before and after the resection of thyroid lobes.^10^,^11^ I-IONM helps nerve identification, determination of neural transmission and loss of signal (LOS). Compared to visual identification, I-IONM also has big advantages to predict the nerve function of a patient who especially has large goitre, locally advanced thyroid cancer, or had a thyroid disease in the past.^12^ Several studies show that using IONM significantly decreases permanent RLN injuries.^8^,^13^

Currently, Intraoperative nerve monitoring is suggested to use for malignant and recurrent pathologies. Because, main reason of RLN injury in these patients is cutting the nerve. On the other hand, in benign thyroid pathologies, main reason is the streching the nerve. But it is not used routinely in primary benign thyroid pathologies. This study presents the effects of intraoperative nerve monitoring on RLN injuries in patients who underwent primary surgery for benign thyroid pathology.

## METHODS

In this study, we retrospectively evaluated the data of 273 patients who had thyroidectomy first time due to benign thyroid pathology between January 2012 and July 2017 at the department of general surgery, Faculty of Medicine, Afyon Health Science University. Patients who had preoperative vocal cord dysfunction, malignant thyroid pathology, recurrent goiters, retrosternal goiters, monitoring problems during the surgery, and the patients who refused to participate the study were excluded from the study. All patients underwent bilateral total thyroidectomy. All patients gave informed consent. Our clinic had intraoperative nerve monitoring in 2015. After that year, the use of nerve monitoring in our clinic become routine. The patients were classified into two groups. Group-1 consists of patients whose nerves were monitored. We separated the patients whose nerves were not monitored into Group-2. Group-2 consisted of patients who were operated before 2015, and Group-1 was operated after 2015. To prevent the effects of the difference of experience between the surgeons, all the patients we included in the study had been operated by the same surgeon. The classified patient groups were analyzed according to age, gender, diagnosis, transient paralysis, permanent paralysis, duration of nerve identification and duration of surgery. We evaluated the effects of I-IONM on transient and permanent RLN injury, duration of nerve identification and duration of surgery. Patients had pre- and post-operative vocal cord examinations. Vocal cord paralysis which continued for more than six months were accepted as permanent and the vocal cord paralysis which continued for less than six months were accepted as transient. Rates of transient and permanent RLN paralysis were compared. For the patients whose RLN was monitored, RLN was protected by going through the trace from the entry of thyroid location beneath the carotid to the entry location of larynx. Intubation was executed without neuromuscular blockage in Group-1. Endotracheal-based visual systems (Dr. Langer Medical, Germany) are used to display the bilateral thyroarytenoid muscles for real time EMG activities. Neural stimulation was done with a disposable probe at 1.5 mA. Before the determination of RLN, an original EMG signal was received from the vagus nerve. Vagal stimulation was applied to discover the availability of tube insertion before the dissection near RLN. Initially, the level of stimulation was adjusted as 1.5 mA and the threshold was 100 mV. When the RLN was found in the tracheo-esophageal gutter, a signal was received from it. The RLN was completely dissected and separated. If the signal was not received at 2 mA, it would be accepted as a failed insertion. After the complete hemostasis in the operating field, the vagus nerve was tested for a last time. Duration of nerve identification was measured as the elapsed time from the received signal with the probe at the RLN identification phase until the nerve being visible and identified with complete dissection.

### Statistical analysis

Data were analysed using SPSS 11.0 for Windows operating system. Results were expressed as mean ± SD. For parametric and nonparametric evaluations, Chi-square and Man-Whitney U tests were used. A P-value less than 0.05 was considered to be significant.

## RESULTS

A total of 140 patients were classified into Group-1. The average age was calculated as 46±11 years old. There were 21 (15%) males and 119 (85%) females. The 119 patients (61.5%) had multinodular goitre, 13 patients (10.8%) had Hashimoto’s thyroid and 8 patients (7.7%) had graves’ disease.

There were 133 patients in Group-2. The average age was calculated as 48±13 years old. There were 17 (12.8%) males and 116 (87.2%) females. A total of 120 patients (65%) had multinodular goitre, nine patients (8%) had Hashimoto’s thyroid and four patients (4.8%) had graves’ disease.

Considering age, gender and surgical indication between the groups, there was no statistically significant difference (P > 0.05) (Table-I). In Group-1, transient paralysis developed in four patients (2.9%). The permanent paralysis developed in one patient (0.7%).

**Table-I:** The features of the groups.

	Group-1 (n=140) IONM (+)	Group-2 (n=133) IONM(-)	P
Age (years old)	46±11	48±13	>0.05
Gender(M/F)	21/119	17/116	>0.05
Surgical indication(n)			
Multinodular Goitre	119	120
Hashimoto	13	9
Graves	8	4

In Group-2, transient paralysis developed in nine patients (6.8%). The permanent paralysis developed in four patients (3%). When the groups were evaluated, there was statistically significant difference in terms of transient and permanent paralysis (P=0.01, P =0.001, respectively). (Table-II).

**Table-II:** Statistical data of the groups according to the complication, duration of nerve identification and duration of surgery.

Complication	Group-1	Group-2	p
Transient paralysis (n)	4(2.9%)	9(6.8%)	0.01
Permanent paralysis (n)	1(0.7%)	4(3%)	0.001
RLN identification duration (min)	5.15 ± 1.3	11.8 ± 2.7	0.001
Duration of surgery (min)	71±14	88±17	>0.05

### Duration of Nerve Identification

Among Group-1, average durations of nerve identification was calculated as 5.15 ± 1.3 minutes. The average durations of nerve identifications in Group-2 was found as 11.8 ± 2.7 minutes. When the durations of nerve identification were evaluated between Group-1 and Group-2, the durations decreased and were found as statistically significant (P=0.001) (Table-II).

### Duration of Surger

Average durations of surgeries among Group-1 was calculated as 71±14 minutes. In Group-2, average durations of surgeries were found as 88±17 minutes. When we evaluated the groups, durations of surgeries in Group-1 were shorter than Group-2 but it was not statistically significant (P > 0.05) (Table-I).

## DISCUSSION

The main reason for vocal cord paralysis which developes after a thyroid surgery is the RLN injury during a thyroidectomy operation. Visibility and protection of RLN in a thyroidectomy operation is the gold standard method to prevent RLN injuries.^6^,^7^ RLN dissection was routinely done first time for each patient by Lahey in 1938. Afterwards, many endocrine surgeons accepted this approach.^14^,^15^ The nerve might be seen with difficultly during dissection when patients particularly are recurrent cases, malignant diseases, anatomic variations and radiation history.^16^-^18^ Discovery and development of nerve monitoring makes the identification of recurrent laryngeal nerves easier.^8^,^17^,^18^ Shedd et al. used the intraoperative nerve monitoring first time in 1966 and it has been developed and increasingly used thus far.^4^,^19^

Using intraoperative nerve monitoring during thyroidectomy allows us to locate the nerve, to determine the anatomic variations and to predict the postoperative functions of vocal cord.^13^-^19^ In case of signal loss during the resection of dominant thyroid lobe, IONM enables the option of gradual thyroidectomy, which reduces the risk of bilateral vocal cord paralysis. This is the biggest advantage of IONM.^5^

On one hand, a few non-randomized studies show that nerve identification with intraoperative nerve monitoring decreases the risk of RLN injury compared to the visual nerve identification.^13^ In a wider non-randomized study with more than 16000 patients in Germany, using intraoperative nerve monitoring reduces the RLN injury risk.^13^,^20^ In a comprehensive multicenter study with 4382 patients, using intraoperative nerve monitoring for the patients operated due to benign goitre decreases the risk of transient and permanent paralysis, which were found statistically significant.^13^-^21^

On the other hand, a one centre study of 1000 patients shows that using intraoperative nerve monitoring does not have benefits for transient and permanent RLN injuries.^17^-^21^ In a prospective study of Sari et al. with 237 patients, a total of 409 recurrent nerves, which were under risk, were examined in two groups.^14^ In Group-1 (n=210), thyroidectomy was done without IONM. In Group-2 (n=199), IONM was performed. Regarding the postoperative complications, no significant difference was found among the groups. Some studies emphasize that an inexperienced surgeon is an independent risk factor. Even for an experienced surgeon, using intraoperative nerve monitoring is fundamental and valuable to decrease the RLN injury risk.^22^

In our study, transient paralysis was found to be 2.9% and 6.8% in Group-1 and 2, respectively. Permanent paralysis was found to be 0.7% and 3%, respectively. When the groups were evaluated, there was statistically significant difference in terms of transient and permanent paralysis (P=0.01, P =0.001, respectively).

In the study of Sopinski et al.^23^ average duration of surgeries was found shorter in the nerve monitoring group (71.29 ± 17.125 minutes and 75.75 ± 17.94 minutes for monitored and non-monitored groups, respectively.) but it was not statistically significant (P=0.377).

Chan et al.^17^ also shows that average surgery duration was shorter in the nerve monitoring group (80 ± 27 minutes and 99 ± 43 minutes for monitored and non-monitored groups, respectively) but it was not statistically significant (P > 0.05).

Our study presents that average durations of surgeries in Group-1 was found as 71 ± 14 minutes. The average durations in Group-2 were 88 ± 17 minutes. Average surgery durations were shorter in the groups that intraoperative nerve monitoring was use, which was in line with the literature. However, this was not statistically significant (P > 0.05).

In the study of Sari et al.^14^, RLN identifications were found shorter and statistically significant (P=0.01) in Group-1 (4.05 ± 1.1 minutes) compared to Group-2 (11.2 ± 2.5 minutes). In our study, average durations of nerve identifications in Group-1 was found as 5.15 ± 1.3 minutes. Average durations in Group-2 was 11.8 ± 2.7 minutes. When the average durations were evaluated according to the groups, shorter durations of nerve identifications were statistically significant (P=0.001).

Shorter durations of nerve identification reduce the surgery duration and stress level of surgeons, although it is thought clinically not very important.^14^ Therefore, IONM during thyroidectomy is prominent for shorter durations of RLN identifications and for lower stress levels of surgeons.^14^ This contributes to reduce the complications while decreasing the stress levels of surgeons.

It is well known that another cause of RLN injury during thyroidectomy operation is stretching the nerve. So, naturally, significantly less duration of nerve identification in the group using intraoperative nerve monitoring reduces the stretching time of the nerve. We think that is why transient and permanent nerve paralysis is less in the group using intraoperative nerve monitoring.

## CONCLUSION

Intraoperative nerve monitoring significantly decreases RLN injury in patients who undergo primary surgery due to benign thyroid pathology. In view of the negative effects of RLN injury on the patient, we think that intraoperative nerve monitoring should be used routinely in benign thyroid surgeries.

### Authors Contribution:

**MA:** Conceived, designed and did statistical analysis & editing of manuscript.

**MA, MC and SY:** Did data collection and manuscript writing.

**YA:** Did review, final approval of manuscript, is responsible for integrity of research.
